# Circadian Plasticity in the Brain of Insects and Rodents

**DOI:** 10.3389/fncir.2018.00032

**Published:** 2018-05-02

**Authors:** Wojciech Krzeptowski, Grzegorz Hess, Elżbieta Pyza

**Affiliations:** ^1^Department of Cell Biology and Imaging, Institute of Zoology and Biomedical Research, Jagiellonian University, Krakow, Poland; ^2^Department of Neurophysiology and Chronobiology, Institute of Zoology and Biomedical Research, Jagiellonian University, Krakow, Poland; ^3^Institute of Pharmacology, Polish Academy of Sciences, Krakow, Poland

**Keywords:** circadian rhythm, circadian clock, synaptic plasticity, neural plasticity, *Drosophila melanogaster*, mice, rat

## Abstract

In both vertebrate and invertebrate brains, neurons, glial cells and synapses are plastic, which means that the physiology and structure of these components are modified in response to internal and external stimuli during development and in mature brains. The term plasticity has been introduced in the last century to describe experience-dependent changes in synapse strength and number. These changes result from local functional and morphological synapse modifications; however, these modifications also occur more commonly in pre- and postsynaptic neurons. As a result, neuron morphology and neuronal networks are constantly modified during the life of animals and humans in response to different stimuli. Nevertheless, it has been discovered in flies and mammals that the number of synapses and size and shape of neurons also oscillate during the day. In most cases, these rhythms are circadian since they are generated by endogenous circadian clocks; however, some rhythmic changes in neuron morphology and synapse number and structure are controlled directly by environmental cues or by both external cues and circadian clocks. When the circadian clock is involved in generating cyclic changes in the nervous system, this type of plasticity is called circadian plasticity. It seems to be important in processing sensory information, in learning and in memory. Disruption of the clock may affect major brain functions.

## Learning and Experience-Dependent Plasticity

Over the last few decades, a wide body of experimental data have demonstrated that the structure and physiology of the nervous system change in response to internal and environmental stimuli. This ability of the nervous system to develop lasting changes in neuron properties that enable adaptation to the environment and regeneration and that underlie the mechanism of memory formation and learning is defined as plasticity. Importantly, changes in neuron morphology, neuronal connectivity, biochemical processes or even new neuron generation are observed during development and also occur widely in the adult nervous system (for review, see Bruel-Jungerman et al., 2007; Sweatt, 2016).

The term “plasticity” was introduced to neurobiology at the beginning of the 20th century (reviewed in Stahnisch and Nitsch, 2002); however, modern theories explaining mechanisms of plasticity of the nervous system have their origin in Hebb’s postulate (Hebb, 1949). The first attempts to describe neural plasticity in relation to learning and memory as well as synaptic plasticity were taken later, and since the late 1960s/early 1970s, the terms “plasticity” and “neuroplasticity” have become common in neurobiology (see Berlucchi and Buchtel, 2009; Fuchs and Flügge, 2014). A breakthrough in understanding synaptic plasticity mechanisms was the discovery that brief trains of electrical stimuli result in increased transmission efficiency at the perforant path-granule cell synapse in the rabbit hippocampus (HC), as measured with electrophysiological recording methods, that can last for hours (Bliss and Lømo, 1973; Lømo, 2003). Over the years, this phenomenon, which was termed long-term potentiation (LTP), has generally been accepted as one of the most popular models of the cellular processes underlying memory (reviewed in Bruel-Jungerman et al., 2007; Sweatt, 2016).

The structural modifications accompanying synaptic plasticity fall into two categories: modifications of existing connections and changes in the number of synapses. Pre-existing synapses are regulated in many ways, such as by the phosphorylation of postsynaptic neurotransmitter receptors or the insertion of additional receptor molecules into postsynaptic membranes, to change their efficiency. On the other hand, the LTP-induced increase in the number of functional synapses was also confirmed in several experiments, and it has been found that changes in the number of synapses are correlated with the reorganization of spine and dendrite morphology (reviewed in Bailey and Kandel, 1993; Kondo, 2017).

Recently, it was reported that LTP induction in the rodent HC varies across a 24 h day both *in vivo* and *in vitro* (Chaudhury et al., 2005; Bowden et al., 2012; Nakatsuka and Natsume, 2014). It is also important to stress that in humans, time-of-day variations have been described for cognitive domains (Schmidt et al., 2007). For example, chronic jet lag (i.e., caused by traveling across time zones) and shift work disrupt the sleep-wake cycle and produce cognitive disturbances (Cho et al., 2000; Rouch et al., 2005).

In this review, we described a special type of neural plasticity, circadian plasticity, that is driven by the circadian clock and synchronized to daily changes in environmental cues. This type of plasticity has been reported in the nervous system of many vertebrate and invertebrate species; however, in this review we focused on rodents and *Drosophila melanogaster* (fruit fly) as the most studied animals in the field of circadian plasticity, including our own studies. Nevertheless, the role of biological clocks in brain plasticity has been reviewed recently by e.g., Frank and Cantera (2014), Iyer et al. (2014), Smarr et al. (2014), Bosler et al. (2015) and Frank (2016).

## Circadian Clock

Circadian rhythms are periodic oscillations that occur within a span of approximately 24 h. They are present in most animal species and are examples of the biological rhythms that have a profound influence on many functions of an organism, ranging from metabolism to complex behaviors. These rhythms are driven by molecular clocks that evolved to synchronize biological processes with time and cyclic changes in the environment. Circadian rhythms allow organisms to predict the coming day and night and to adapt to different conditions during the day in advance. Although the molecular components of the clock are not conserved across the main divisions of life, the molecular mechanism of the circadian clock in most species is based on negative and positive transcriptional–translational feedback loops, in which transcription factors activate the expression of their own repressors (reviewed i.e., by Rosbash, 2009). Importantly, this molecular clock is composed of core clock genes/proteins and regulates the rhythmic expression of other genes (so-called clock-controlled genes, *ccg*s) in various tissues (Doherty and Kay, 2010).

### *Drosophila* Clock Neurons

**Figure 1:**
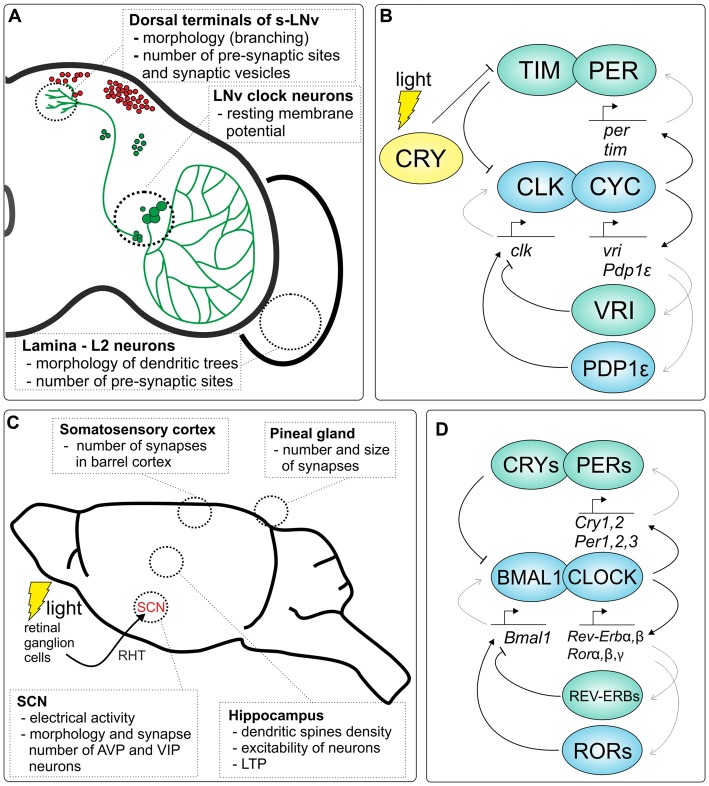
Clock-dependent plasticity in the brain of *Drosophila* and rodents. **(A)** Examples of circadian plasticity in the *Drosophila* brain. The localization of DN1–3 clock neurons in the brain is shown in red, while lateral clock neurons and their pigment-dispersing factor (PDF)-positive projections are depicted in green. **(B)** Molecular mechanism of the fruit fly circadian clock. Two major transcriptional/translational feedback loops are shown, which are regulated by the CLOCK/CYCLE (CLK/CYC) dimer, TIMELESS/PERIOD (TIM/PER) and VRILLE/PAR DOMAIN PROTEIN 1ε (VRI/PDP1ε) loops (activators are marked in blue, while inhibitors are green). Light-activated CRYPTOCHROME (CRY) protein binds TIM to initiate proteasomal degradation which allows to synchronize the clock to external light conditions. **(C)** A schematic representation of the rodent brain with examples of circadian plasticity in different structures of the brain. The clock in the suprachiasmatic nucleus (SCN) is synchronized by light through the retina; mainly photosensitive retinal ganglion cells (melanopsin cells) are involved in clock light perception via the retinohypothalamic tract (RHT). **(D)** Molecular clock in mammals. Two major transcriptional/translational feedback loops, PER/CRY and REV-ERB/ROR, are regulated by the CLOCK/BMAL1 dimer (activators are marked in blue, while inhibitors are green).

In insects, most of the early work on circadian rhythms and clocks has been done on large non-model species such as cockroaches and crickets (Nishiitsutsuji-Uwo and Pittendrigh, 1968; Page, 1982; Tomioka, 1985). Nevertheless, the clock has been best characterized in *D. melanogaster*, a common model organism in genetics, development and neurobiology. Approximately 150 neurons, in the fruit fly’s brain, have been identified as clock neurons (Figure 1A), based on the cyclic expression of so-called clock genes and clock proteins (described in Section “Molecular mechanisms of the circadian clock”). These cells are located in distinct clusters and are divided into seven major groups, based on their anatomical position, comprising three groups of dorsal neurons (DN; DN1–3) and four groups of lateral neurons (LNds, LPNs, l-LNvs and s-LNvs). The large and small ventral lateral neurons (l-LNvs and s-LNvs, respectively) are the best-studied clock neurons of *D. melanogaster*, as they express pigment-dispersing factor (PDF), a neurotransmitter important for clock neuron internal synchronization and for the output pathways of the fly’s circadian clock (Kaneko and Hall, 2000; Helfrich-Förster, 2003, 2005; Rieger et al., 2006; Yoshii et al., 2008; Damulewicz and Pyza, 2011; Damulewicz et al., 2013; Beckwith and Ceriani, 2015).

### Circadian Clock in Mammals

Regarding the structure in the mammalian brain responsible for maintaining circadian rhythms, transplantations (Aguilar-Roblero et al., 1986; Lehman et al., 1987; Ralph et al., 1990; Sollars et al., 1995; Sujino et al., 2003) and surgical lesions (Moore and Eichler, 1972; Stephan and Zucker, 1972; Mouret et al., 1978) of the hypothalamus have shown that the primary circadian oscillator is located in the ventral periventricular zone of the hypothalamus, in the suprachiasmatic nucleus (SCN; reviewed in Buhr and Takahashi, 2013). The direct synchronization of this circadian clock with environmental changes in light and darkness (LD) is possible by photosensitive retinal ganglion cells via the retinohypothalamic tract (RHT), which is the retinal projection terminating in the SCN (Hendrickson et al., 1972; Moore and Lenn, 1972; reviewed in Hughes et al., 2015; Figure 1C). Initially, it was believed that the SCN is the only structure capable of integrating environmental cues and driving internally sustained rhythms of cellular activity and gene expression, which is why it was named the “master clock” or “pacemaker”. However, recent studies have revealed that peripheral oscillators located in other tissues in the body are also enmeshed within the regulation of numerous metabolic pathways. These local clocks are able to generate the rhythms even when they are separated from the brain. Thus, recently, it is believed that the SCN does not initiate but rather synchronizes different peripheral oscillators throughout the body to a uniform time (Buhr and Takahashi, 2013).

### Molecular Mechanisms of the Circadian Clock

The core molecular components that underlie the generation of circadian oscillations in animals are best described in mammals and the fruit fly (Figures 1B,D). The current model of the clock at the molecular level is based on two main interconnected transcriptional/translational feedback loops that function together to generate cyclic gene expression. At the heart of the molecular clock, CLOCK (or its analog NPAS2) and BMAL1 in mammals, and CLOCK (CLK) and CYCLE (CYC) in *Drosophila* have been identified as transcriptional activators.

In *Drosophila* CLK and CYC form heterodimers and activate the expression of *period* (*per*) and *timeless* (*tim*) which encode their own repressors PER and TIM proteins in the primary feedback loop. CLK/CYC heterodimers also regulate their own expression via the second loop by the activation of *vrille (vri)* and *Par domain protein 1ε (Pdp1ε)* transcription. Both VRI and PDP1ε proteins bind to the promoter region of *Clk*, however VRI inhibits, while PDP1ε activates, *Clk* transcription. In addition, these transcriptional feedback loops are additionally regulated posttranslationally by numerous cytoplasmic proteins, e.g., kinases, that together generate endogenous circadian rhythms at the level of mRNAs and proteins of the molecular oscillator. In addition, CLK/CYC regulates the expression of other genes, so-called *ccg*s, which drive circadian rhythms in physiological and cellular processes. The circadian clock of flies also possesses its own photoreceptor, the CRYPTOCHROME (CRY) that is involved in light entrainment.

In mammals, in the first loop, after dimerization and translocation to the nucleus, CLOCK/BMAL1 initiates the transcription of *Per1, Per2, Per3* and* Cry1, Cry2* encoding their own repressors: PERs and CRYs. In the second loop, CLOCK/BMAL1 regulates their own transcription by controlling the expression of two other genes, *Rorα/β/γ* and *Rev-erbα/β*, that encode retinoid acid receptor-related orphan receptors (RORs) and orphan nuclear receptors (REV-ERBs), respectively. Both, RORs and REV-ERBs regulate the expression of *Bmal1*; however, RORs act as activators while REV-ERBs are repressors. As in *Drosophila* CLOCK/BMAL1 also regulates the expression of *ccg*s.

For the most recent and more detailed reviews on the molecular mechanism of the circadian clock in mammals, see Buhr and Takahashi (2013), and for the fly’s clock, see Özkaya and Rosato (2012) or Tataroglu and Emery (2015).

The existence of output pathways of the circadian clock enables the regulation of many genes. Genome-wide studies have identified numerous rhythmically expressed genes in non-neuronal tissues (Storch et al., 2002) and the nervous system. In the human brain (Li et al., 2013) and different regions of the mouse brain, including the HC (Renaud et al., 2015), prefrontal cortex (Yang et al., 2007), cerebellum, hypothalamus and brain stem (Zhang et al., 2014), dozens of genes exhibit daily rhythms of transcriptional activity. Significant differences in the level of many transcripts during the day and night have also been identified in the cerebral cortex (Cirelli et al., 2004) and in the rat pineal gland (Fukuhara and Tosini, 2008; Bailey et al., 2009). Diurnal and circadian expression of several genes has also been confirmed in insect heads (Claridge-Chang et al., 2001; McDonald and Rosbash, 2001; Ceriani et al., 2002; Ueda et al., 2002; Wijnen et al., 2006; Keegan et al., 2007; Naeger et al., 2011; Rodriguez-Zas et al., 2012). Interestingly, many of these genes are involved in remodeling neuronal circuits and/or synapses and might be directly involved in the regulation of brain plasticity.

## *Drosophila Melanogaster* as a Model Species to Study Circadian Plasticity in the Brain

The fruit fly’s brain, despite its simplicity, shows pronounced plasticity, being continuously remodeled in response to environmental stimuli and by intrinsic circadian inputs (summarized in Figure 1A). Due to a large number of available genetic tools and the high homology of genes and proteins between *Drosophila* and mammals, this species is exceptionally useful in research on circadian rhythm and sleep, as well as complex behaviors such as learning and memory, and in research on circadian plasticity (Heisenberg et al., 1995; Greenspan and Dierick, 2004; Olsen and Wilson, 2008; Pyza, 2013).

### Clock Neuron Circadian Plasticity

Neurons that comprise the central clock not only regulate the circadian plasticity in the brain but also undergo circadian remodeling of their own processes. These morphological changes, besides the circadian release of PDF (Park et al., 2000), contribute to the downstream transmission of information. Indeed, the dorsal terminals of s-LN_v_s are more complex in the morning than during the early night. These changes are maintained in constant darkness (DD) but are abolished in *per*^01^ and *tim*^01^ clock mutants, confirming their circadian origin (Fernández et al., 2008). Recently, it has been found that the retraction of s-LN_v_ axonal terminals at dusk depends on actin and myosin light-chain phosphorylation, which in turn is regulated by clock-controlled Rho1 protein activity (Petsakou et al., 2015). Circadian oscillations in the morphology of neurites have also been detected in the housefly‘s clock neurons (Pyza and Meinertzhagen, 1997), while daily structural changes have been observed in the PDF-immunoreactive fibers of the cockroach *Leucophaea maderae* (Wei and Stengl, 2011).

Circadian changes in the morphology of PDF-positive fruit fly neurons are accompanied by changes in the number of synapses. The number of active zones labeled by antibody against Bruchpilot, a presynaptic scaffolding protein, and the number of vesicles detected by anti-Synaptotagmin serum are increased at the beginning of the subjective day in DD. Moreover, s-LN_v_s seem to form synaptic contacts with different partners, which allows these neurons to regulate different processes in the brain during the day and night. These results revealed that circadian network activity in the brain can be regulated not only by modulating synapse strength between different cells but also by changes in the number of synaptic contacts between particular groups of neurons (Gorostiza et al., 2014).

Clock neuron plasticity is accompanied by rhythmic changes in cell membrane excitability that are critical for maintaining their function. Electrical silencing of the pacemaker neurons by targeting K+ channel expression inhibits the clock and leads to arrhythmic behavior (Nitabach et al., 2002). On the other hand, whole-cell recordings from a large subset of LN_v_s have shown that their resting membrane potential is modulated by both the clock and light input (Park and Griffith, 2006). Other studies also confirmed the circadian origin of membrane excitability, as well as the pattern and action potential firing rate of lateral ventral clock neurons (Cao and Nitabach, 2008; Sheeba et al., 2008).

The circadian remodeling of s-LN_v_ terminals and the ability of s-LN_v_s to change axonal arbor conformation in response to neuronal firing requires myocyte enhancer factor 2 (Mef2), and the *Mef2* gene is a direct target of the master circadian regulator dimer CLK/CYC. Therefore, it seems that circadian information is transmitted to the machinery involved in neuronal remodeling via the Mef2 protein (Sivachenko et al., 2013). Interestingly, in the mammalian nervous system, MEF2 suppresses the number of excitatory synapses and thus regulates the strength of neuronal connections (Flavell et al., 2006).

### Circadian Plasticity in the Visual System

Since the function of cells in the visual system of *D. melanogaster* is similar to the function of cells in the retina of vertebrates, the visual system of the fruit fly is a good model for studying various processes, e.g., circadian rhythms, at the cellular level (Pyza, 2002). The visual system of *D. melanogaster*, as in other insect species, is composed of the retina and three optic neuropils: the lamina, medulla and lobula complex. Circadian changes in neuronal morphology in the nervous system were first described by Pyza and Meinertzhagen (1993, 1995) in the housefly (*Musca domestica*), the blowfly (*Calliphora vicina*, Pyza and Cymborowski, 2001) and also in the fruit fly (Pyza and Meinertzhagen, 1999).

**Figure 2:**
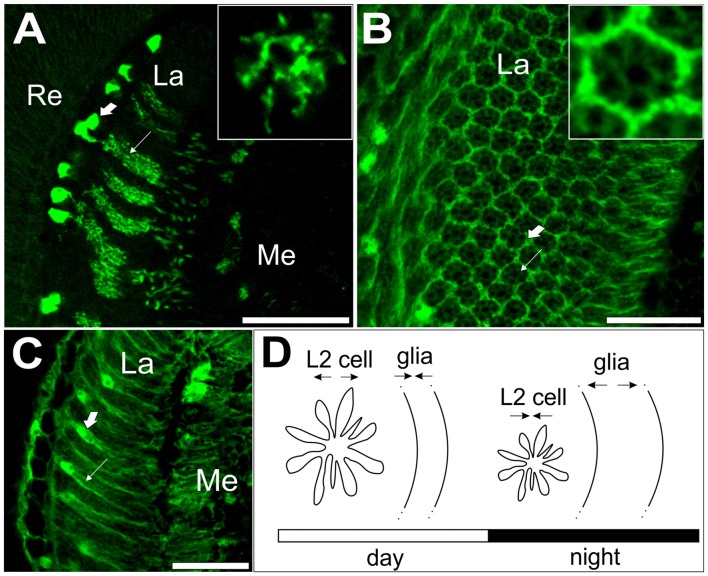
L2 interneurons and glial cells showing circadian plasticity in the visual system of *Drosophila melanogaster*. **(A)** GFP-labeled L2 monopolar cells, first order interneurons, in the optic lobe lamina. Large arrow indicates L2 somata; small arrow indicates L2 axons with dendrites. Insert: cross section of an L2 axon with dendrites. **(B)** Cross section of the lamina with GFP-labeled epithelial glial cells (large arrow) surrounding lamina cartridges (small arrow). Insert: a single cartridge surrounded by three epithelial glial cells. **(C)** GFP-labeled epithelial glial cells in the lamina. Large arrow indicates the soma; small arrow indicates processes of epithelial glial cells. **(D)** A schematic representation of circadian plasticity in the L2 dendritic tree and epithelial glial cells. Changes in neuron size are offset by changes in glia size. Re, retina, La, lamina, Me, medulla of the optic lobe. Scale bar: 20 μm (Pyza and Górska-Andrzejak, 2004; Weber et al., 2009).

For example, in the retina of *D. melanogaster*, the enzyme hemeoxygenase that metabolizes heme to carbon monoxide, ferrous iron and biliverdin affects the molecular mechanism of the circadian clock in photoreceptors (Damulewicz et al., 2017). However, the most robust circadian rhythms have been described in the lamina. The cross-sectional area of L1 and L2 monopolar cells, the first order interneurons in the lamina, increases twice a day: in the morning and in the evening (Pyza and Meinertzhagen, 1999). Similarly, analysis of GFP-labeled L2 cells in the lamina revealed the daily changes in the L2 dendrite length, with longest dendrites at the beginning of both day and night (Figures 2A,D; Górska-Andrzejak et al., 2005). Rhythmic changes in the size of the L2 dendritic tree are controlled by the circadian clock since the rhythm is still present in constant darkness, while in the null *per*^01^ clock mutants, the rhythm is abolished, and the dendrites are shorter than those in wild-type flies. Moreover, in *cry*^b^ mutants, the morning peak of the L2 dendritic tree perimeter is shifted, which indicates that CRY is important for setting the phase of the circadian oscillation in L2 dendrite morphology (Weber et al., 2009). The involvement of core clock genes in neuronal morphology regulation has also been described for s-LN_v_s. In *tim*^01^ mutants, the axons of these neurons are shorter and hyperbranched, while in *per*^01^ they are longer with less complicated branching (Fernández et al., 2008).

Similar to the circadian plasticity of the PDF-positive neurons of the central clock, changes in the morphology of non-clock interneurons in the visual system of *D. melanogaster* seem to be correlated with the circadian oscillation in the number of synapses formed between photoreceptors and postsynaptic cells, including L1 and L2 interneurons. In the lamina, two types of synapses, tetrad and feedback, have been examined during the 24 h cycle. Tetrad synapses, formed between the photoreceptor terminal R1-R6 and four postsynaptic cells, constitute the majority of synapses in the lamina, while feedback synapses, between L2 back onto R1-R6, are less numerous. The presynaptic profiles of tetrad synapses exhibit a bimodal rhythm, with two peaks in the morning and in the evening, whereas the feedback synapse frequency peaks at night (Woźnicka et al., 2015). Remodeling of these synaptic contacts is correlated with daily or circadian fluctuations in synaptic protein levels, such as Bruchpilot (Górska-Andrzejak et al., 2013), Synapsin and Disc large (Krzeptowski et al., 2014).

Structural circadian plasticity in the visual system of the fruit fly is modulated by PDF and ion transport peptide (ITP) produced by the 5th small LN_v_ that projects to the lamina (Damulewicz and Pyza, 2011; Damulewicz et al., 2013, 2015). Moreover, it appears that clock-controlled activity in the *D. melanogaster* visual system is regulated by a mechanism based on Na^+^/K^+^-ATPase (Górska-Andrzejak et al., 2009; Damulewicz et al., 2013, 2015). This type of clock-controlled regulation has also been described in the mammalian SCN (Wang and Huang, 2004).

### Circadian Changes in Motor Terminals

Another example of circadian-dependent neural plasticity described in *D*. *melanogaster* has been found in the MN5 motor neuron that innervates two longitudinal indirect flight muscles. In flies reared in LD conditions, the size of MN5 terminal boutons is significantly larger in the middle of the day than in the middle of the night, and this rhythm is maintained in constant darkness, which proves its regulation by the circadian clock. These findings have been confirmed in *per* and *tim* clock gene mutants, which lacked daily changes in the size of MN5 boutons (Mehnert et al., 2007). Similar to the results obtained studying PDF-positive s-LNv terminals (Fernández et al., 2008), *per*^01^ mutants presented with significantly fewer branches than wild-type flies, while the *tim* mutation causes hyperbranching of axonal projections. This type of plasticity in *D. melanogaster* seems to be regulated locally by a peripheral clock located in the thoracic ganglia, as the rhythm remains intact in decapitated flies (Mehnert and Cantera, 2008). The most recent study revealed rhythmic changes in the number of motor neuron synapses in both LD and DD conditions and light-dependent mechanisms in the regulation of synapse number throughout the day (Ruiz et al., 2013), similar to those discovered in the lamina (Górska-Andrzejak et al., 2013; Woźnicka et al., 2015).

### Memory Formation in *D. melanogaster*

It has been reported that short-term associative memory formation is modulated by the circadian clock in *Drosophila*. Lyons and Roman (2008) found that a particular time of day improves memory formation and the performance peak occurs during the early night/subjective night in both LD and DD. Moreover, this rhythm is absent in *per*^01^ and *tim*^01^ mutants as well as in constant light conditions (LL); however, this rhythm is present in *cry*^b^ flies. These results indicate that the central clock, not peripheral oscillators or the perception of stimuli, regulates the formation of short-term associative memory (Lyons and Roman, 2008). Such modulation of memory formation might be controlled by the s-LN_v_s that have projections near the calyx of mushroom bodies, which are memory-processing structures in the insect’s brain (Heisenberg, 1998; Helfrich-Förster et al., 2002).

## Cyclic Plasticity in the Mammalian Brain

Several studies have demonstrated that rapid changes in the efficiency of existing synapses and modifications to already formed neural networks are important for nervous system plasticity in mammals. This process can be regulated, among other factors, by environmental light/dark cycles and by the endogenous circadian clock. Importantly, these daily or circadian structural changes in the adult nervous system have been described not only in the SCN but also in other regions of the brain, e.g., the HC, and in the retina (summarized in Figure 1C).

### Circadian Plasticity of the SCN

As mentioned above, the SCN is a master clock in mammals that synchronizes circadian peripheral clocks throughout the body and, like in the fruit fly, undergoes daily activity and structure reorganization. In the SCN, circadian rhythms in neural activity seem to be crucial for proper central clock function (Brown and Piggins, 2007; Colwell, 2011). Multiunit *in vivo* recordings of diurnal electrical activity patterns in the rat SCN and experiments conducted in constant darkness (Inouye and Kawamura, 1979, 1982), as well as the spontaneous firing rate of rat SCN neurons recorded *in vitro* in hypothalamic slice preparations (Green and Gillette, 1982; Groos and Hendriks, 1982; Shibata et al., 1982), have demonstrated the circadian nature of the mentioned rhythms.

Indeed, rhythmic oscillations of the membrane potential in the SCN are essential for neuropeptide release and thus contribute to clock-based plasticity (reviewed in Iyer et al., 2014). Recent studies focused on the rearrangement of two main sources of the SCN efferents: neurons expressing arginine vasopressin (AVP) and vasoactive intestinal peptide (VIP). Becquet et al. (2008) reported a decrease in the mean coverage of somata and VIP neuron dendrites during night-time and, conversely, an increase in the extent of somata and dendritic membrane appositions involving AVP neurons. In turn, the results obtained by Girardet et al. (2010) showed that synaptic contacts on neurons expressing VIP increase during the day but AVP neuron connections remain unchanged. These data are similar to those observed in the clock neurons of *Drosophila* (described in the previous paragraph) and support the hypothesis that the SCN structure undergoes circadian plasticity, which is necessary for the central clock to synchronize with the LD cycle.

### Circadian Changes in the Hippocampus

The SCN is connected directly and indirectly to many brain regions, including the HC, a structure that is crucial for learning and memory formation. Early *in vivo* experiments performed by Barnes et al. (1977), revealed that basal synaptic transmission, measured as the amplitude of the excitatory postsynaptic potential (EPSP) evoked in the rat dentate gyrus by afferent stimulation, is larger during the night. In contrast to rats, which are nocturnal animals, monkeys exhibit a larger response amplitude during the day. On the other hand, West and Deadwyler (1980) did not find circadian variations in dentate gyrus synaptic currents in rats, but they reported that the amplitude of the population spikes elicited by perforant path axon stimulation is greater during the light phase of the LD cycle. Importantly, these changes were not induced by the intensity of the behavioral arousal or circulating corticosterone level (West and Deadwyler, 1980), indicating that the excitability of granule cells may vary in a circadian-dependent manner.

Harris and Teyler (1983) found that in the rat hippocampal CA1 area, LTP is evoked more readily during the day than during the night, whereas the dentate gyrus develops more robust LTP during the dark phase of the LD cycle. In the CA1 area of hippocampal slices from Syrian hamsters, more robust LTP was also detected during the day (Raghavan et al., 1999). However, in mice, LTP in the CA1 hippocampal area was significantly greater during the night (Chaudhury et al., 2005). The variability in these data is likely a result of differences in experimental approaches, including the recording of either field EPSPs or population spikes and different afferent stimulation paradigms (Nakatsuka and Natsume, 2014). *In vivo* recording results from rat CA1 pyramidal cells suggest that firing rates go through a sinusoidal cycle on a time scale with a 25-h period (Munn and Bilkey, 2012). This may be related to the observed diurnal differences in LTP magnitude in CA1 when population spike amplitudes are measured. Moreover, recently, it was found that the suppression of local inhibitory connections during the dark phase contributes to larger LTP during that phase (Nakatsuka and Natsume, 2014).

The circadian regulation of neural plasticity is associated with fluctuations in neuron morphology and synaptic contact remodeling. Dendritic spines, carrying postsynaptic elements of synapses, regulate the synaptic strength of neuronal networks. The process of dendritic spine generation and elimination is dynamic and occurs even without any specific environmental stimulation, and spine density fluctuates during the day, as was observed in the Siberian hamster HC (Ikeno et al., 2014). Diurnal changes in the spine density of CA1 pyramidal neurons were also detected *in vivo* with fixed rat HC, and compared with the sleep state, the highest density was observed upon waking (Ikeda et al., 2015). Interestingly in the mouse HC, adult neurogenesis occurs under the control of clock genes (Bouchard-Cannon et al., 2013; Schnell et al., 2014).

### Neural and Synaptic Clock-Dependent Plasticity in Other Parts of the Brain

Other brain regions also appear to be regulated by the circadian clock. For example, dendritic morphology of rat infralimbic cortex neurons changes with diurnal phases of resting and activity. During the animal active period, dendrites are longer and have more spines than during the resting period (Perez-Cruz et al., 2009).

**Figure 3:**
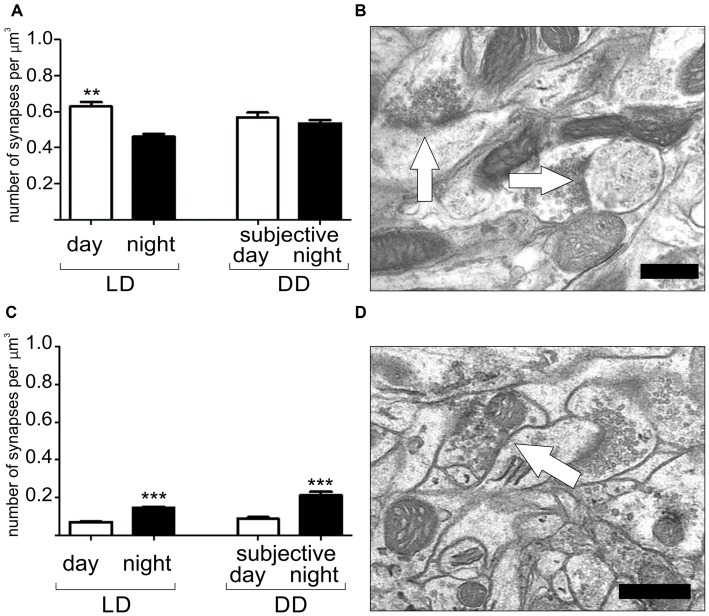
Daily and circadian rhythms in the number of excitatory **(A,B)** and inhibitory synapses **(C,D)** in the barrel cortex of the mouse somatosensory cortex. Light and darkness (LD) 12:12–12 h of light and 12 h of darkness, DD–constant darkness. Scale bar: 0.5 μm. Graphs show means ± SD, **P < 0.01, ***P < 0.001 (Jasinska et al., 2015).

Robust circadian plasticity has been observed in the somatosensory cortex of mice (Figure 3). During the night, when mice are active, sensory input signals, received by whiskers, are transmitted to barrels in the fourth layer of the somatosensory cortex, the whisker representation region. Under LD conditions, the number of excitatory synapses in the barrels is highest when mice are resting (light phase of the LD cycle), while inhibitory synapse density increases during their locomotor activity (dark phase of the LD cycle). Inhibitory synapse density is controlled endogenously by the circadian clock, as the robust rhythm of the number of this type of synapse is also maintained in constant darkness. In contrast, the number of excitatory synapses is influenced by light because their density is constant during both active and rest times in animals kept in DD. These results indicate that the sensory input associated with the circadian rhythm of animal rest and activity regulates daily and circadian rhythms in synapse density in the mouse somatosensory cortex (Jasinska et al., 2014, 2015). In addition to the circadian rhythm in synapse density, Jasinska et al. (2015) have also reported changes in the number of single and double synapse dendritic spines. The increased number of double synapse spines during the dark phase of the LD cycle suggests that, during the night, inhibitory synapses can be added to existing dendritic spines carrying one excitatory synapse.

In vertebrates, cyclic plasticity also concerns so-called ribbon synapses in the pineal gland, the retina and other sensory organs. Analyses of ribbon size by both transmission electron microscopy and fluorescent labeling of synaptic proteins revealed that ribbon length varies in LD conditions. In the pineal gland of rats or guinea pigs, ribbon number and size increase during the night (Vollrath, 1973; Jastrow et al., 1997), while mouse photoreceptor synaptic ribbons also change their shape during the day/night cycle but exhibit the reverse pattern, being longest during the day under LD conditions (Adly et al., 1999; Balkema et al., 2001). Striking changes in the number and shape of ribbon synapses indicate that the number and morphology of these synapses are remodeled during the day.

Circadian synaptic plasticity has also been described in other vertebrate species (Elbaz et al., 2013). For example, in zebrafish larvae, the number of presynaptic elements in hypocretin/orexin (HCRT) neurons exhibits circadian rhythmicity (Appelbaum et al., 2010), and circadian rhythmicity in the synaptic ribbons of goldfish pineal glands was described by McNulty (1981).

## Mechanisms of Circadian Plasticity of the Brain

The mechanisms of clock-based brain plasticity are not yet fully recognized. Circadian changes observed in the nervous system probably rely on intracellular fluctuations in circadian clock proteins, the activity of enzymes in clock cells and circadian changes in the expression of small molecules known to participate in neural plasticity that are controlled by the central and peripheral clock system. Each of these molecules might be regulated in different ways.

In mammals, the expression of core clock genes has been confirmed not only in the SCN but also in other brain structures, including the cortex and HC (Namihira et al., 1999; Reick et al., 2001; Shieh, 2003; Jilg et al., 2010; Ono et al., 2015; Riddle et al., 2016; Yamaguchi et al., 2016). Moreover, several studies have shown that clock gene mutations disrupt synaptic plasticity, memory formation and learning. For example, *Per1* knockout mice display defects in LTP recorded *in vivo* (Rawashdeh et al., 2014) and in the HC-dependent long-term spatial learning paradigm (Jilg et al., 2010), while *Per2* mutant mice exhibit abnormal hippocampal LTP and deficits in trace recall (Wang et al., 2009). Mutations in other core clock genes also affect brain plasticity, as *Npas2* mutant mice show impairments in cued and contextual fear memory (Garcia et al., 2000), while *Cry1*^−/−^ *Cry2*^−/−^ mice fail to learn time-place associations, and their electroretinogram is changed, indicating abnormalities in retinal visual processing (Cameron et al., 2008; Van der Zee et al., 2008). In turn, Kondratova et al. (2010) found alterations in short- and long-term memory formation in *Bmal1*^−/−^ mutants, while Wardlaw et al. (2014) found defects in learning and spatial memory and a reduced LTP magnitude in hippocampal slices from the same mouse strain.

The results obtained by Rawashdeh et al. (2014) highlighted another important issue, namely, daytime deficits in the working memory performance of* Per1* knockout mice, which was correlated with a loss of CREB phosphorylation circadian rhythm in the HC. Interestingly, hippocampal plasticity is modulated by effectors also involved in SCN plasticity, such as CREB, BDNF and γ-aminobutyric acid (GABA; Girardet et al., 2013; Schildt et al., 2013; Martin-Fairey and Nunez, 2014; Nakatsuka and Natsume, 2014; Hwang et al., 2016; Rawashdeh et al., 2016; Albers et al., 2017). For example, hippocampal-based learning seems to be strictly correlated with the circadian-controlled regulation of mitogen-activated protein kinase (MAPK) cascade activity, which is crucial for inducing hippocampal LTP and the formation of long-term memory. Moreover, circadian oscillations in cAMP and MAPK activity in the HC are absent in memory-deficient mice that lack calmodulin-dependent adenylyl cyclase activity. In general, physiological or pharmacological disruptions in hippocampal MAPK activity oscillations impair memory persistence (Eckel-Mahan et al., 2008; Eckel-Mahan, 2012).

The existence of common molecular pathways in the SCN and other brain structures, such as the HC, make it difficult to determine whether changes in brain plasticity and learning are regulated by the SCN or by local peripheral clocks. Some results suggest that SCN lesions abolish rhythms of passive avoidance in rats (Stephan and Kovacevic, 1978; Ruby et al., 2008). Furthermore, in arrhythmic Siberian hamsters, the daily variance in novel object learning is abolished (Ruby et al., 2008). However, the most recent reports suggest that the SCN is not necessary for learning. For example, in golden hamsters, after the removal of the SCN, the circadian modulation of conditioned place preference was unaffected, which suggests an extra-SCN origin of the rhythm (Cain and Ralph, 2009). This conclusion is supported by the presence of cyclic *Per2* gene expression in the isolated hippocampal slices of those animals (Wang et al., 2009). In turn Storch et al. (2007) showed that a lack of *Bmal1* expression in the retina leads to the disruption of retinal electrical responses to light, similar to results in *Bmal1*^−/−^ mutant mice. Finally, circadian plasticity regulation by peripheral clocks has also been confirmed in the structure of MN5 motor neurons in *D. melanogaster* (Mehnert and Cantera, 2008). On the other hand, in the HC, the circadian-based regulation of long-term memory via the cAMP/MAPK/CREB transcriptional pathway seems to be maintained by the SCN, as electrolytic lesions of this structure ablate circadian oscillations in hippocampal MAPK pathway activation and lead to long-term memory deficits (Phan et al., 2011). In addition, circadian oscillations in glucocorticoid activity under normal physiological conditions are correlated with the formation and stabilization of new dendritic spines in the HC (Liston et al., 2013). These results indicate that the circadian clock in the SCN may regulate only specific aspects of brain plasticity and that local oscillators also contribute to this process.

Indeed, over the past few years, it has become clear that the central clock (pacemaker) in the brain is a part of the larger network of clocks, including peripheral clocks, which interact with each other and with many other systems within the organism. The coordination of rhythmicity among different cells is a complicated process in which many factors seem to be involved. These neurons might communicate with each other by synaptic and paracrine mechanisms and/or by gap junctions. In the fruit fly, PDF plays a major role in the synchronization and modulation of oscillations between clock neurons and in the transmission of circadian information downstream from clock neurons. It is also possible that additional mechanisms are involved in maintaining circadian rhythms since disrupting fast synaptic transmission in PDF-positive clock cells does not affect the circadian rhythms in behavior (Renn et al., 1999; Kaneko et al., 2000; Park et al., 2000; Blanchardon et al., 2001; Johard et al., 2009; Im and Taghert, 2010; Yao and Shafer, 2014; reviewed also in Muraro et al., 2013; Beckwith and Ceriani, 2015). In the SCN, vasointestinal peptide and other peptides are involved in circadian signaling (Harmar, 2003; O’Neill and Reddy, 2012). Interestingly, despite the same clock core genes and proteins existing in the SCN and peripheral clocks, which are involved in maintaining circadian oscillations, the regulation of their expression might be slightly different. For example, the *Per1* and *Per2* mRNA levels are controlled by transcription factors other than CLOCK/BMAL1, and their activity is under the control of systemic signals, such as hormones, temperature and secondary messengers [(Travnickova-Bendova et al., 2002; So et al., 2009; Hanyu et al., 2011; Tamaru et al., 2011; Chappuis et al., 2013), reviewed also in an excellent article on circadian synaptic plasticity (Frank, 2016)].

The circadian multioscillatory system is even more complicated since glial cells have also been proposed to act as circadian oscillators in both mammals (Prolo et al., 2005; Yagita et al., 2010) and insects (Siwicki et al., 1988; Zerr et al., 1990; Ewer et al., 1992; Suh and Jackson, 2007). Recently, it has been confirmed by qPCR methods that *per* and *tim* genes have rhythmic expression in the glial cells of fruit flies (Damulewicz et al., 2015). Glial cells seem to also be involved in the regulation of neuron circadian plasticity. In the housefly lamina, epithelial glial cells undergo daily changes in morphology (Figures 2B–D; Pyza and Górska-Andrzejak, 2004). More interestingly, glial metabolism disruption or blocking gap junctions affects circadian plasticity in L2 interneurons (Pyza and Górska-Andrzejak, 2004), while disrupting the clock in glia changes the daily pattern of BRP expression in the lamina (Górska-Andrzejak et al., 2013). In mammals, it has been suggested that the circadian clock mechanism within microglia surrounding synapses drives the circadian modulation of dendritic spine density and synaptic strength via cathepsin S (Hayashi et al., 2013). While a detailed description of the role of glia in the regulation of circadian plasticity is outside the scope of this review, readers are encouraged to read the following reviews: Jackson (2011), Jackson et al. (2015) and Chi-Castañeda and Ortega (2016).

## Conclusion

During day and night, the brain undergoes significant functional and morphological changes, and some of these rhythms are generated by endogenous mechanisms driven by circadian clocks. These cyclical changes in the efficiency and number of synapses or in the morphology of neurons and glial cells seem to be correlated with locomotor activity in both vertebrates and invertebrates. However, the most common problem in interpreting the results of experiments on circadian-controlled brain functions is the fact that it is not always obvious if the observed rhythm is clock- or state-driven. Recently, a “state-clock” model, which integrates clock- and state-driven changes in synaptic plasticity, has been introduced. In this model, clock outputs regulate circadian plasticity processes, and global changes observed across sleep and wake cycles are driven by clocks and not by the state of the brain (Frank and Cantera, 2014; Frank, 2016). Nevertheless, future studies are necessary to better understand these interactions and the regulation of circadian plasticity in the brain.

## Author Contributions

WK, GH and EP contributed to the writing, literature review and revision of this article. EP proposed the concept of the manuscript.

## Conflict of Interest Statement

The authors declare that the research was conducted in the absence of any commercial or financial relationships that could be construed as a potential conflict of interest. The reviewer SS and handling Editor declared their shared affiliation.
